# Rearrangement of mitochondrial tRNA genes in flat bugs (Hemiptera: Aradidae)

**DOI:** 10.1038/srep25725

**Published:** 2016-05-16

**Authors:** Fan Song, Hu Li, Renfu Shao, Aimin Shi, Xiaoshuan Bai, Xiaorong Zheng, Ernst Heiss, Wanzhi Cai

**Affiliations:** 1 Department of Entomology, China Agricultural University, Beijing 100193, China; 2 GeneCology Research Centre, Faculty of Science, Health, Education and Engineering, University of the Sunshine Coast, Maroochydore, Queensland 4556, Australia; 3 College of Life Sciences and Technology, Inner Mongolia Normal University, Hohhot 010022, China; 4 Department of Plant Pathology and Crop Protection, Georg-August-University Göttingen, Göttingen 37077, Germany; 5 Tiroler Landesmuseum, Josef-Schraffl-Strassbe 2a, A-6020 Innsbruck, Austria

## Abstract

The typical insect mitochondrial (mt) genome organization, which contains a single chromosome with 37 genes, was found in the infraorder Pentatomomorpha (suborder Heteroptera). The arrangement of mt genes in these true bugs is usually the same as the ancestral mt gene arrangement of insects. Rearrangement of transfer RNA (tRNA) genes, however, has been found in two subfamilies of flat bugs (Mezirinae and Calisiinae, family Aradidae). In this study, we sequenced the complete mt genomes of four species from three other subfamilies (Aradinae, Carventinae and Aneurinae). We found tRNA gene rearrangement in all of these four species. All of the rearranged tRNA genes are located between the mitochondrial control region and *cox1*, indicating this region as a hotspot for gene rearrangement in flat bugs; the rearrangement is likely caused by events of tandem duplication and random deletion of genes. Furthermore, our phylogenetic and dating analyses indicated that the swap of positions between *trnQ* and *trnI* occurred ~162 million years ago (MYA) in the most recent common ancestor of the five subfamilies of flat bugs investigated to date, whereas the swap of positions between *trnC* and *trnW* occurred later in the lineage leading to Calisiinae, and the translocation of *trnC* and *trnY* occurred later than 134 MYA in the lineage leading to Aradinae.

Mitochondrial (mt) genomes are one of the most explored sources of molecular markers for studying animal phylogeny and phylogeography^1^,^2^. In addition to the sequences of mt genes and mt genomes, arrangement of mt genes has also been explored in these studies^3^,^4^,^5^. Like in most other animals, insect mt genomes are circular and contain 37 genes (13 protein-coding genes, two ribosomal RNA genes, and 22 transfer RNA genes) and a control region on a single chromosome^2^,^6^. The arrangement of genes in mt genomes is usually stable in insects. Indeed, most insects known retained the ancestral mt gene arrangement of Pancrustacea (i.e. hexapods and crustaceans)^6^,^7^. However, multipartite and fragmented mt genomes with two or more chromosomes or minichromosomes have been found in sucking lice (order Phthiraptera)^8^, booklice (Order Psocoptera)^9^,^10^ and thrips (order Thysanoptera)^11^. Extensive rearrangement of mt genes has also been found in these three paraneopteran orders^9^,^12^,^13^,^14^.

The ancestral mt gene arrangement of insects is retained in most species of the fourth paraneopteran order, Hemiptera^15^,^16^,^17^,^18^; however, gene rearrangement has been found in the suborder Sternorrhyncha (i.e. whiteflies)^19^,^20^. True bugs (suborder Heteroptera), with the largest number of published complete mt genomes in Hemiptera, showed mt gene rearrangement in unique-headed bugs^21^, pyrrhocoroid bugs^22^ and flat bugs^22^,^23^,^24^. Flat bugs (family Aradidae) is a relatively large family in the infraorder Pentatomomorpha of Heteroptera, with approximately 1,970 species and 270 genera in eight extant subfamilies and a extinct subfamily Archearadinae^25^,^26^,^27^. Most of the flat bugs are mycophagous (i.e. fungi eater) and are generally found under tree bark^28^. The sister relationship between Aradidae and Termitaphididae (together comprising the superfamily Aradoidea) is less controversial and the Aradoidea is consistently placed as sister to the rest of Pentatomomorpha^29^. However, the phylogenetic relationships among the subfamilies of flat bugs are still poorly studied^27^,^30^.

Prior to this study, the mt genomes of three flat bugs have been sequenced, representing two subfamilies: *Neuroctenus parus* and *Brachyrhynchus hsiaoi* from the subfamily Mezirinae^22^,^24^, and *Aradacanthia heissi* from the subfamily Calisiinae^23^. All of these three species have tRNA gene rearrangement near the control region in their mt genomes. To understand how tRNA gene rearrangement occurred in the flat bugs (family Aradidae), we sequenced the mt genomes of four more species of flat bugs from three other subfamilies: *Aradus compar* (Aradinae), *Libiocoris heissi* (Carventinae), *Aneurus similis* (Aneurinae) and *Aneurus sublobatus* (Aneurinae). We compared the mt gene arrangement among the seven species of flat bugs from five subfamilies of the Aradidae. Our results indicated that mt tRNA gene rearrangement in flat bugs was driven by events of tandem duplication and random deletion near the control region. *trnQ* and *trnI* swapped their positions in the most recent common ancestor of the five subfamilies of flat bugs ~162 million years ago (MYA); later, *trnC* and *trnW* swapped their positions in the lineage leading to the subfamily Calisiinae whereas *trnC* and *trnY* were translocated in the lineage leading to the subfamily Aradinae later than 134 MYA.

## Results

### General features of flat bug mitochondrial genomes

**Figure 1:**
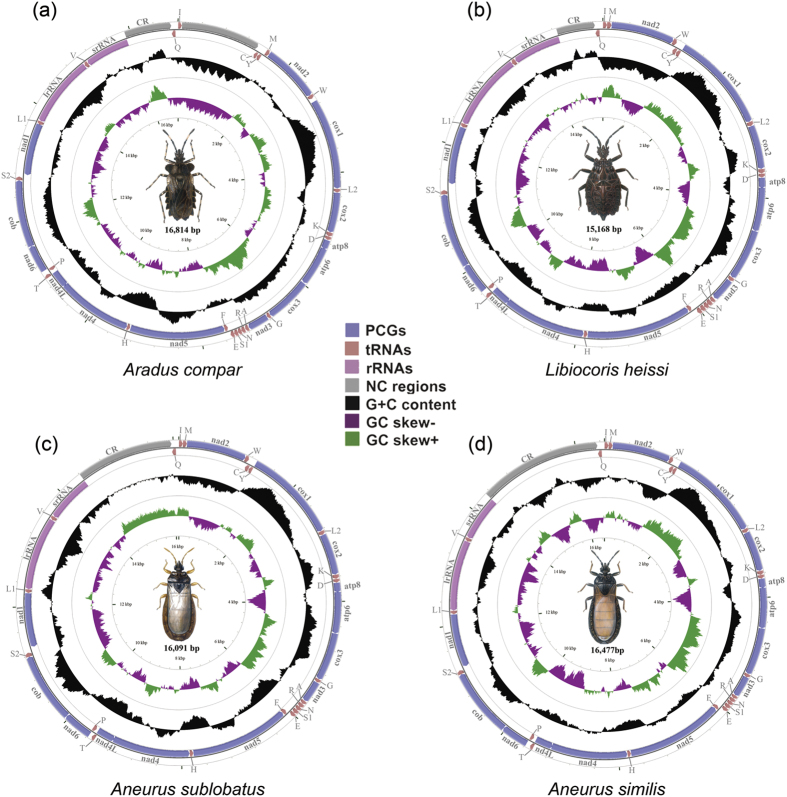
Mitochondrial genomes of four flat bugs sequenced in this study. Circular maps were drawn with CGView^66^. Arrows indicate the orientation of gene transcription. Abbreviations of gene names are: *atp6* and *atp8* for ATP synthase subunits 6 and 8, *cox1*–*3* for cytochrome oxidase subunits 1–3, *cob* for cytochrome b, *nad1*–*6* and *nad4L* for NADH dehydrogenase subunits 1–6 and 4L, *lrRNA* and *srRNA* for large and small rRNA subunits. tRNA genes are indicated with their one-letter corresponding amino acids. CR for control region. The GC content was plotted using a black sliding window, as the deviation from the average GC content of the entire sequence. GC-skew was plotted as the deviation from the average GC-skew of the entire sequence. The illustrations of four flat bugs were drawn by F.S.

We sequenced the complete mt genomes of four species of flat bugs in the present study (Fig. 1), making mt genome sequences available for seven species of flat bugs representing five subfamilies of the Aradidae (Table 1). The mt genomes of the seven flat bugs range from 15,168 bp (*Libiocoris heissi*) to 16,814 bp (*Aradus compar*) in length. Each mt genome of the flat bug contains the 37 genes commonly found in animal mt genomes, as well as a putative control region (CR) which included the presumed origin of replication and promoters for transcription initiation^6^. All of the protein coding-genes (PCGs) start with ATN codons except for *cox1*, which start with TTG codon (Supplementary Table S1).

The mt genomes of the flat bugs were AT biased and showed the positive AT- and negative GC-skews (Supplementary Table S2), as is usually observed in insect mt genomes^31^. For PCGs, genes on the J-strand had a positive AT- and negative GC-skews, whereas the opposites were observed on the N-strand. It has been proposed that the strand bias of nucleotide composition might be related to replication and transcription mechanisms^32^.

### Control regions and intergenic spacers

**Figure 2:**
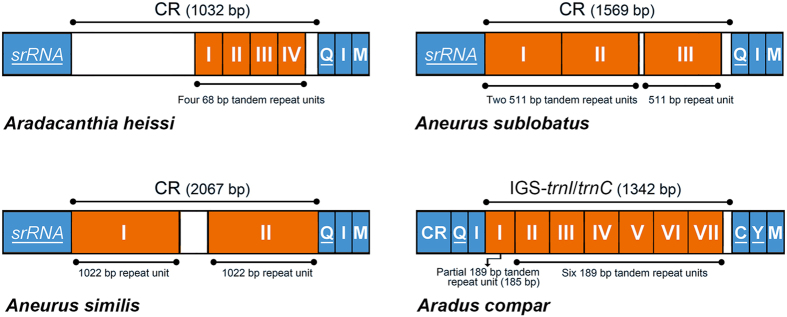
Repeat sequences in the control region and the intergenic spacer of flat bug mitochondrial genomes. Abbreviations of gene names follow Fig. 1. Genes are transcribed from left to right except those underlined, which have the opposite transcriptional orientation. The location and copy number of repeat units were shown by colored rectangle with Roman numerals inside. IGS-*trnI*/*trnC* for the intergenic spacer between *trnI* and *trnC*.

The CRs of the seven flat bugs varied considerably in length, ranging from 586 bp in *Libiocoris heissi* to 2,067 bp in *Aneurus similis*. Repeat sequences were found in the CRs of *Aradacanthia heissi*, *Aneurus similis* and *Aneurus sublobatus*: four copies of 68-bp tandem repeat unit in *Aradacanthia heissi*, two identical 1,022-bp repeats in *Aneurus similis*, and three identical 511-bp repeat units in *Aneurus sublobatus* (the first two units are tandem repeats) (Fig. 2).

In addition to the CRs, we also identified three intergenic spacers (IGS), between the genes *trnQ*-*trnI*, *trnS*-*nad1*, and *trnP*-*nad6* that we designate “IGS-*trnQ*/*trnI*, IGS-*trnS2*/*nad1* and IGS-*trnP*/*nad6*”, respectively, in all of the seven flat bug mt genomes (Table S3). Two long IGSs, between the genes *trnI*-*trnC* and *trnW*-*cox1* that we designate “IGS-*trnI*/*trnC* and IGS-*trnW*/*cox1”*, were found in *Aradus compar*. Tandem repeat sequences were also found in IGS-*trnI*/*trnC* (1,342 bp) in *Aradus compar* (Fig. 2). Most of the IGSs were located between rearranged genes, e.g. IGS-*trnQ*/*trnI* (44–135 bp) in all of the seven species and IGS-*trnI*/*trnC* and IGS-*trnW*/*cox1* (56 bp) in *Aradus compar* (discussed below). In general, the CRs and IGSs varied substantially in both length and sequence among the seven species of flat bugs.

### Gene rearrangement

**Figure 3:**
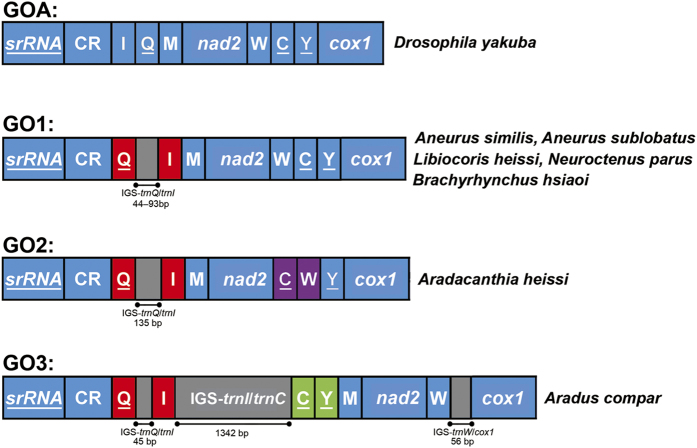
Mitochondrial gene rearrangements in flat bugs. Abbreviations of gene names follow Fig. 1. Genes are transcribed from left to right except those underlined, which have the opposite transcriptional orientation. Rearrangements of tRNA genes were highlighted by color. IGS for the intergenic spacer.

Compared with the ancestral mt gene order of insects (GOA, Fig. 3)^6^, tRNA gene rearrangement was found in all of the seven flat bugs and resulted in three types of gene orders (GO1–3). The five species from the subfamilies Aneurinae, Carventinae and Mezirinae possessed the same gene order (GO1) with the swap of positions between *trnQ* and *trnI* (Fig. 3). GO2 was found in *Aradacanthia heissi* with the swap of positions between *trnQ* and *trnI*, and between *trnC* and *trnW* (Fig. 3). The swap of positions between *trnQ* and *trnI* and the translocation of *trnC* and *trnY* were found in *Aradus compar* (GO3; Fig. 3).

### Phylogenetic relationship and divergence time reconstruction

To understand the evolution of tRNA gene rearrangement in flat bugs, we performed phylogenetic analyses and estimated the divergence time for Aradidae and Pentatomomorpha. Twenty-seven Pentatomomorpha species from five superfamilies (Aradoidea, Pentatomoidea, Lygaeoidea, Pyrrhocoroidea and Coreoidea), together with six outgroup species from Cimicomorpha, were included in our phylogenetic analyses. We used maximum likelihood (ML) and Bayesian inference (BI) methods and generated four phylogenetic trees with two data matrices, PCGR and PCG12R (also see Materials and Methods below). The monophyly of each superfamily was well supported in all trees with bootstrap values (BS) 100 and posterior probabilities (PP) 1 (Supplementary Fig. S1). The sister relationship between Aradoidea and other superfamilies was also highly supported (BS = 100 and PP = 1). Pentatomoidea was placed as the sister group to Eutrichophora (Lygaeoidea, Pyrrhocoroidea and Coreoidea). In Eutrichophora, we found conflicting phylogenetic results between different datasets and methods. The sister-group relationship between Pyrrhocoroidea and Lygaeoidea was recovered in two BI trees (PP = 1 and PP = 0.99) and ML analysis of the dataset PCGR (BS = 73), however, ML analysis of the dataset PCG12R supported the sister group of Pyrrhocoroidea and Coreoidea (BS = 49). The relationships among the five subfamilies of flat bugs were recovered as: (Calisiinae + (Aradinae + (Mezirinae + Carventinae + Aneurinae))). Although the relationship among the three subfamilies (Mezirinae, Carventinae and Aneurinae) was not well resolved (BS < 65 and PP < 0.62) based on the present taxon sampling, the close relationship of these three subfamilies to the exclusion of other two subfamilies was strongly supported in both ML and BI analyses (BS = 100 and PP = 1).

**Figure 4:**
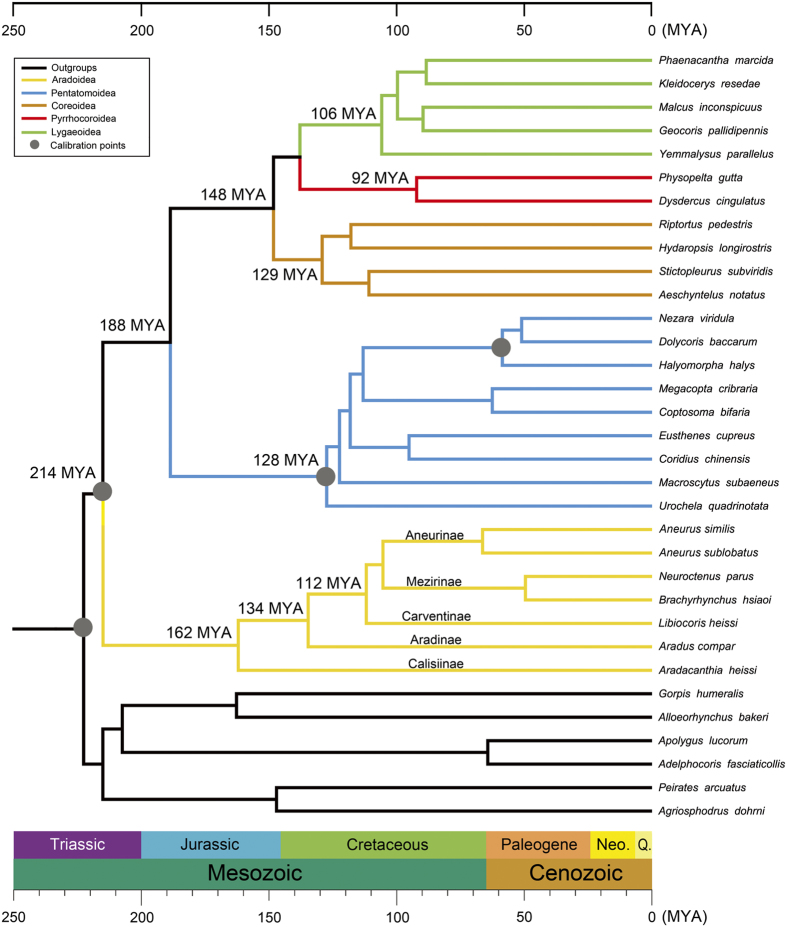
Chronogram showing Pentatomomorpha phylogeny and divergence time. Consensus tree presenting divergence dates produces by the PhyloBayes analysis of the PCG12R dataset using four fossil calibration points (Supplementary Table S9), the autocorrelated Lognormal relaxed-clock model, the site-heterogeneous mixture CAT + GTR substitution model, and soft bound 10%. A geological time scale is shown at the bottom.

Relaxed molecular clock analyses were performed for our two datasets using four fossil calibration points. Results were not significantly different between the two datasets (PCG12R and PCGR; Supplementary Table S4). The most recent common ancestor (MRCA) of Pentatomomorpha was estimated to be at the late Triassic (~214 MYA); the diversification into five superfamilies occurred from the late Jurassic to middle Cretaceous (214 to 92 MYA) (Fig. 4). The MRCA of flat bugs (Aradidae; five of the eight extant subfamilies included in the present study) was dated to be ~162 MYA (confidence interval, 181 to 140 MYA). Aradinae separated from Carventinae, Mezirinae and Aneurinae at around 125 MYA (PCGR) and 134 MYA (PCG12R).

## Discussion

Flat bugs (family Aradidae) are a relatively large family in Pentatomomorpha, with eight extant subfamilies and approximately 1,970 species^25^,^26^; the monophyly of Aradidae is accepted generally^27^. The seven species of flat bugs, for which mt genomes have been sequenced, have three types of tRNA gene rearrangement. Our phylogenetic and molecular clock analyses of the mitochondrial genome sequences indicated that the swap of positions between *trnQ* and *trnI* is shared by all seven species of flat bugs and thus likely occurred in their MRCA ~162 MYA. *trnC* and *trnW* swapped their positions later in the lineage leading to the subfamily Calisiinae, and *trnC* and *trnY* were translocated in the lineage leading to the subfamily Aradinae later than 134 MYA. Divergence times of the main lineages of the flat bugs estimated in our analyses are consistent with their fossil records, for example Aradinae separated from Carventinae, Mezirinae and Aneurinae in early Cretaceous, which was in accordance with the oldest Aradinae fossils *Aradus nicholasi* (125 to 113 MYA)^33^; and the diversification between the extinct subfamily Archearadinae (represented by two species, *Archearadus burmensis*^34^,^35^ and *Microaradus anticus*^36^) and the extant subfamilies also took place in early Cretaceous (~100 MYA)^37^. Our study provided a preliminary framework for understanding the evolution of the flat bugs and their mitochondrial genomes. Further studies with more complete sampling are needed to reconstruct a comprehensive phylogeny and more accurate molecular dating.

Of the several mechanisms proposed to explain mt gene rearrangement, tandem duplication followed by random gene loss (TDRL) is generally considered the most important in insects^38^. According to the TDRL, duplication of part of the mt genome was caused by the slipped-strand mispairing or inaccurate termination during replication and novel gene orders generated from random deletion of the supernumerary gene copies^3^,^5^. Alternative mechanisms including inversion^39^, tandem duplication/nonrandom loss (TDNR)^40^, tRNA duplication/anticodon mutation^41^,^42^,^43^, and recombination^44^,^45^ have been proposed to account for mt gene rearrangements that cannot be explained by TDRL model alone.

**Figure 5:**
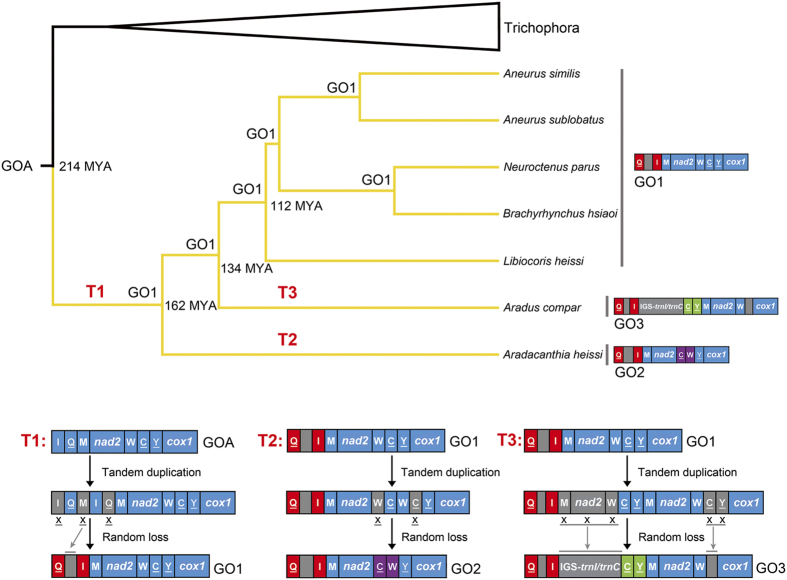
Reconstruction of the mitochondrial rearrangement scenarios in the evolution of flat bugs and proposed mechanism of tRNA gene rearrangement in the model of TDRL. Phylogenetic tree were simplified from Fig. 4. Genes are transcribed from left to right except those underlined, which have the opposite transcriptional orientation. Rearrangements of tRNA genes were highlighted by color. IGS for the intergenic spacer. Eliminated genes were indicated by crosses and pointed to the relative IGSs by grey arrows.

When mapped on the phylogenetic tree, it was clear that GO1 was an ancestral character for flat bugs and occurred ~162 MYA via a TDRL event (T1: from *trnI*-*trnQ*-*trnM* to *trnQ*-*trnI*-*trnM*) from GOA in the MRCA of Pentatomomorpha. T1 event could be simply a TDRL of *trnI* and *trnQ* (from *trnI*-*trnQ*-*trnI*-*trnQ* to *trnQ*-*trnI*). However, the observed IGS-*trnQ*/*trnI* in GO1 could not be explained by such process (Fig. 3). The mt genome retained one copy of duplicated genes during subsequent evolution, the other copy was deleted or became pseudogenes or IGSs^5^,^46^,^47^. So, we assumed that the gene cluster of *trnI*-*trnQ*-*trnM* was duplicated in tandem (*trnI*-*trnQ*-*trnM*-*trnI*-*trnQ*-*trnM*) and one copy of each duplicated gene was randomly deleted during T1 event (Fig. 5). The second TDRL event (T2: from *trnW*-*trnC*-*trnY* to *trnC*-*trnW*-*trnY*) changed the GO1 to GO2 in the subfamily Calisiinae. No IGS was found between *trnC* and *trnW* in GO2 so T2 event was likely caused by tandem duplication of *trnW* and *trnC* followed by random loss of one copy of each duplicated gene. The GO3 of Aradinae was derived from GO1 via another TDRL event (T3: *trnM*-*nad2*-*trnW*-*trnC*-*trnY* to *trnC*-*trnY*-*trnM*-*nad2*-*trnW*). The IGSs between *trnI* and *trnC*, *trnW* and *cox1* in *Aradus compar* which corresponds to random losses of genes, provided evidence supporting the steps of gene duplication and deletion in the gene rearrangement from GO1 to GO3.

All of the tRNA gene rearrangement occurred in the region between CR and *cox1*. Many studies have noted that tandem duplications and gene deletions may be subject to mechanistic constraints such that genes flanking the origins of strand replication (e.g., the CR) are more likely to be duplicated, forming “hotspots” of gene rearrangement that make convergent gene order rearrangement more probable^5^,^48^. Various rearrangements of this hotspot region have been found in many insects, e.g., almost all species of Hymenoptera have the rearranged position of *trnI* and *trnQ*^48^; all species of the Lepidoptera suborder Ditrysia have the arrangement *trnM*-*trnI*-*trnQ*^49^; and most species of Neuroptera have the transposition of *trnW* and *trnC*^50^,^51^.

## Materials and Methods

### Samples and DNA extraction

The flat bugs, *Aneurus similis*, *Aneurus sublobatus*, *Aradus compar* and *Libiocoris heissi* were collected in China, and the sampling information was provided in Supplementary Table S5. Specimens were initially preserved in 100% ethanol in the field and transferred to −20 °C for long-term storage at the Entomological Museum of China Agricultural University (Beijing, China). For each species, the genomic DNA was extracted from one adult’s muscle tissues of the thorax using the DNeasy DNA Extraction kit (QIAGEN).

### Genome sequencing, assembly and annotation

The mt genomes were amplified and sequenced as described in Li *et al*.^15^,^21^. Protein-coding genes and two rRNA genes were identified by BLAST searches in NCBI (http://www.ncbi.nlm.nih.gov) and then confirmed by alignment with homologous genes from closely related species. tRNA genes were identified using the tRNAscan-SE^52^ and ARWEN^53^ and checked manually. Two tRNAs (*trnR* and *trnS1)* not found by the programs were determined based on similarities with sequences from closely related species. The annotated mt genome sequences of four flat bugs have been deposited in GenBank under accession numbers: JQ780816, JQ780817, JQ780818 and JQ780819. The nucleotide composition of each mt genome was calculated using MEGA 6.0^54^. We also measured the AT- and GC-skews for whole mt genome sequences and protein-coding genes.

### Sequence alignment and phylogenetic analyses

We retrieved nucleotide sequences of the 13 mt protein-coding genes and two rRNA genes for 29 species of insects from the NCBI. To this initial data set, we added mt genomes of the four flat bugs determined in the current study, thus generating a dataset of 33 taxa (27 Pentatomomorpha species and six Cimicomorpha species as outgroups, see Supplementary Table S6).

Each protein-coding gene was aligned individually based on codon-based multiple alignments using the MAFFT algorithm within the TranslatorX^55^ online platform. Poorly aligned sites were removed from the protein alignment before back-translate to nucleotides by using GBlocks within the TranslatorX with default settings. Sequences of each rRNA gene were individually aligned using the MAFFT v7.0 online server with G-INS-i strategy^56^. Ambiguous positions in the alignment of rRNAs were filtered using GBlocks v0.91b^57^ with default settings. Two datasets were assembled for phylogenetic analyses: 1) the PCGR matrix, including all three codon positions of PCGs and two rRNA genes (total of 12,231 bp); 2) the PCG12R matrix, including the first and the second codon positions of PCGs and two rRNA genes (total of 8,783 bp). The optimal partition strategy and models of each dataset was selected by PartitionFinder v1.1.1^58^. We created an input configuration file that contained 15 pre-define partitions by gene. We used the “greedy” algorithm with branch lengths estimated as “unlinked” and Akaike Information Criterion (AIC) to search for the best-fit scheme (Supplementary Table S7). All these datasets were analyzed under maximum likelihood (ML) framework by using RAxML- HPC2 8.1.11^59^. Bootstrapping analysis with 1,000 replicates was performed with the fast ML method implemented in RAxML using GTRGAMMA model for nucleotide data.

A 10-fold Bayesian cross-validation was performed to test the fit of the site-heterogeneous mixture models (CAT and CAT + GTR) and “site-homogeneous” models (GTR) to nucleotide dataset using PhyloBayes 3.3 f^60^ (see PhyloBayes manual). The result showed that the CAT + GTR model was the best fitting model for both datasets (data not shown). We then inferred phylogenies from the PCG12R and PCGR datasets using PhyloBayes MPI 1.4 f^61^, with the CAT + GTR model. In each individual analysis, two independent chains starting from a random tree were run for 30,000 cycles, with trees being sampled every cycle until 30,000 trees were sampled. The initial 7,500 trees of each MCMC run were discarded as burn-in. A consensus tree was computed from the remaining 45,000 trees combined from two runs.

### Divergence time estimation

Divergence time was estimated for each of the two nucleotide datasets using PhyloBayes 3.3 f^60^, using the best fitting relaxed clock models and the tree generated from the PCG12R dataset and CAT + GTR model. We used Bayes factor (calculated using thermodynamic integration) in PhyloBayes to compare three widely used relaxed models (Lognormal, CIR, and UGAM)^62^. Bayes factor analysis was conducted by running 10,000 points, sampling every 10 after a burn-in of 1,000. The uncorrelated UGAM model fall into the same category as the models implemented in BEAST^63^, and this model is shown to fit the data more poorly than two autocorrelated models (CIR and Lognormal). As the Bayes factors for the CIR and Lognormal models were similar, therefore “-auto” analyses (see PhyloBayes manual) were used to compare these two models. Results of Bayes factors are shown in Supplementary Table S8.

For all molecular clock analyses, a birth-death prior on divergence time and fossil calibrations with soft bounds^64^ were used. Following the recommendations of Benton and Donoghue^65^, minimum constraints were taken as the upper boundary (youngest) of the time period suggested for the oldest fossil in a crown clade, and maximum as the lower boundary (oldest) of the time period suggested for the nearest well-preserved plesiomorphic relative of the clade. Considering the conflicting relationships among Pyrrhocoroidea, Lygaeoidea and Coreoidea in phylogenetic analyses, we only selected four fossil calibrations for Pentatomidae, Pentatomoidea, Pentatomomorpha and the split of Pentatomomorpha and Cimicomorpha (Supplementary Table S9). The range of fossil age was collected from relevant literature on fossils and a recent version of the Paleobiology Database (https://paleobiodb.org/). We allocated 10% of the probability mass to lie outside each calibration interval. All calculations were performed by running 20,000 generations and sampled every 10 generations (after burn-in of 2,000 generations).

## Additional Information

**How to cite this article**: Song, F. *et al*. Rearrangement of mitochondrial tRNA genes in flat bugs (Hemiptera: Aradidae). *Sci. Rep*. **6**, 25725; doi: 10.1038/srep25725 (2016).

## Supplementary Material

Supplementary Information

## Figures and Tables

**Table 1:** Structural features of flat bug mitochondrial genomes.

Subfamily	Species	Whole genome		*lrRNA*		*srRNA*		Control region		Source
		Size(bp)	A + T(%)	Size(bp)	A + T%	Size(bp)	A + T%	Size(bp)	A + T%	
Mezirinae	*Neuroctenus parus*	15354	68.9	1514	71.9	785	68.8	649	69.2	NC_012459
	*Brachyrhynchus hsiaoi*	15250	70.4	1245	74.2	808	72.5	703	69.8	NC_022670
Calisiinae	*Aradacanthia heissi*	15528	74.7	1230	77.6	788	74.0	1032	81.5	HQ441233
Aradinae	*Aradus compar*	16814	64.1	1267	69.8	801	62.7	795	64.5	present study
Carventinae	*Libiocoris heissi*	15168	68.7	1242	72.0	782	70.1	586	66.9	present study
Aneurinae	*Aneurus sublobatus*	16091	67.7	1251	70.3	790	68.9	1569	68.1	present study
	*Aneurus similis*	16477	68.0	1234	71.6	789	68.9	2067	69.5	present study
