# Supplementary material for: Rearrangement of mitochondrial tRNA genes in flat bugs (Hemiptera: Aradidae)
Source: Sci Rep. 2016 May 16;6:25725. doi: 10.1038/srep25725 (PMC4867608; doi:10.1038/srep25725)
Supplement: Supplementary Information [file srep25725-s1.doc]

**Rearrangement of mitochondrial tRNA genes in flat bugs (Hemiptera: Aradidae)**

Fan Song^1*^, Hu Li^1*§^, Renfu Shao^2^, Aimin Shi^1^, Xiaoshuan Bai^3^, Xiaorong Zheng^1,4^, Ernst Heiss^5^, Wanzhi Cai^1§^

^1^Department of Entomology, China Agricultural University, Beijing, China

^2^GeneCology Research Centre, Faculty of Science, Health, Education and Engineering, University of the Sunshine Coast, Maroochydore, Queensland 4556, Australia

^3^College of Life Sciences and Technology, Inner Mongolia Normal University, Hohhot, China

^4^ Department of Plant Pathology and Crop Protection, Georg-August-University Göttingen, Göttingen 37077, Germany

^5^Tiroler Landesmuseum, Josef-Schraffl-Strabe 2a, A-6020 Innsbruck, Austria

^*^These authors contributed equally to this work.

§Correspondence and requests for materials should be addressed to H. L. (tigerleecau@hotmail.com) and W. C. (caiwz@cau.edu.cn).

**Supplementary Information**

**Supplementary Table S1. Codon distribution in flat bug mitochondrial genomes.**

| **Protein-coding genes** | ***Neuroctenus parus*** | ***Brachyrhynchus hsiaoi*** | ***Aradacanthia heissi*** | ***Aradus compar*** | ***Libiocoris heissi*** | ***Aneurus sublobatus*** | ***Aneurus similis*** |
| --- | --- | --- | --- | --- | --- | --- | --- |
| *cox1* | 1,536 | 1, 536 | 1, 539 | 1, 536 | 1, 534 | 1, 536 | 1,535 |
|  | (TTG/TAA) | (TTG/TAG) | (TTG/TAA) | (TTG/TAG) | (TTG/T-) | (TTG/TAA) | (TTG/TA-) |
| *cox2* | 673 | 673 | 679 | 679 | 670 | 673 | 670 |
|  | (ATA/T-) | (TTG/T-) | (ATA/T-) | (ATA/T-) | (ATA/T-) | (ATA/T-) | (ATA/T-) |
| *cox3* | 783 | 781 | 784 | 781 | 787 | 784 | 787 |
|  | (ATG/TAA) | (ATG/T-) | (ATG/T-) | (ATA/T-) | (ATG/T-) | (ATG/T-) | (ATG/T-) |
| *cob* | 1,132 | 1,134 | 1,137 | 1,137 | 1,134 | 1,137 | 1,137 |
|  | (ATG/T-) | (ATG/TAA) | (ATG/TAG) | (ATG/TAA) | (ATG/TAA) | (ATG/TAG) | (ATG/TAG) |
| *nad1* | 933 | 921 | 936 | 930 | 927 | 918 | 927 |
|  | (ATT/TAA) | (TTG/TAA) | (ATA/TAG) | (GTG/TAA) | (TTG/TAA) | (TTG/TAG) | (TTG/TAA) |
| *nad2* | 976 | 981 | 987 | 981 | 969 | 972 | 969 |
|  | (ATA/T-) | (ATA/TAA) | (ATA/TAG) | (ATA/TAA) | (ATA/TAA) | (ATA/TAA) | (ATT/TAA) |
| *nad3* | 353 | 354 | 352 | 354 | 351 | 354 | 352 |
|  | (ATA/TA-) | (ATA/TAG) | (ATA/T-) | (ATA/TAA) | (ATT/TAA) | (ATA/TAA) | (ATA/T-) |
| *nad4* | 1,318 | 1, 309 | 1,326 | 1,335 | 1,332 | 1,325 | 1,332 |
|  | (ATG/T-) | (ATG/T-) | (ATG/TAA) | (ATT/TAG) | (ATG/TAA) | (ATG/TA-) | (ATG/TAG) |
| *nad4L* | 291 | 291 | 294 | 306 | 297 | 309 | 306 |
|  | (ATT/TAA) | (ATT/TAA) | (ATT/TAA) | (ATT/TAG) | (ATT/TAA) | (ATT/TAA) | (TTG/TAA) |
| *nad5* | 1,684 | 1,687 | 1,681 | 1,693 | 1,684 | 1,692 | 1,687 |
|  | (ATT/T-) | (ATG/T-) | (ATT/T-) | (ATA/T-) | (ATT/T-) | (ATA/TAG) | (ATT/T-) |
| *nad6* | 453 | 456 | 444 | 468 | 468 | 477 | 459 |
|  | (ATA/TAA) | (ATT/TAA) | (ATA/TAA) | (ATT/TAA) | (TTG/TAA) | (ATT/TAA) | (ATA/TAA) |
| *atp6* | 669 | 669 | 664 | 669 | 669 | 672 | 672 |
|  | (ATG/TAA) | (ATG/TAA) | (ATG/T-) | (ATG/TAA) | (ATG/TAA) | (ATG/TAA) | (ATG/TAA) |
| *atp8* | 144 | 153 | 156 | 159 | 159 | 156 | 159 |
|  | (ATA/TAA) | (ATA/TAA) | (ATC/TAA) | (ATC/TAA) | (ATC/TAA) | (ATA/TAA) | (ATA/TAA) |

**Supplementary Table S2.** AT and GC-skews of flat bug mitochondrial genomes.

|  |  | **Whole genome** | | **Protein-coding genes** | | **Protein-coding genes-J** | | **Protein-coding genes-N** | |
| --- | --- | --- | --- | --- | --- | --- | --- | --- | --- |
| **Subfamily** | **Species** | **AT-Skew** | **GC-Skew** | **AT-Skew** | **GC-Skew** | **AT-Skew** | **GC-Skew** | **AT-Skew** | **GC-Skew** |
| Mezirinae | *Neuroctenus parus* | 0.20 | -0.22 | -0.09 | -0.03 | 0.10 | -0.20 | -0.40 | 0.27 |
|  | *Brachyrhynchus hsiaoi* | 0.16 | -0.18 | -0.09 | -0.04 | 0.07 | -0.17 | -0.34 | 0.20 |
| Calisiinae | *Aradacanthia heissi* | 0.16 | -0.16 | -0.09 | -0.02 | 0.07 | -0.13 | -0.35 | 0.19 |
| Aradinae | *Aradus compar* | 0.18 | -0.20 | -0.13 | -0.02 | 0.07 | -0.17 | -0.43 | 0.23 |
| Carventinae | *Libiocoris heissi* | 0.23 | -0.28 | -0.10 | -0.04 | 0.13 | -0.25 | -0.46 | 0.34 |
| Aneurinae | *Aneurus sublobatus* | 0.20 | -0.23 | -0.12 | -0.07 | 0.12 | -0.24 | -0.48 | 0.26 |
|  | *Aneurus similis* | 0.21 | -0.28 | -0.11 | -0.06 | 0.12 | -0.25 | -0.46 | 0.30 |

**Supplementary Table S3.** Statistics on intergenic spacers in flat bug mitochondrial genomes.

| **Subfamily** |  | ***trnQ-trnI*** | ***trnP-nad6*** | ***trnS2-nad1*** | ***trnI-trnC*** | ***trnW-cox1*** |
| --- | --- | --- | --- | --- | --- | --- |
| Mezirinae | *Neuroctenus parus* | 44 | 2 | 18 | - | - |
|  | *Brachyrhynchus hsiaoi* | 93 | 2 | 21 | - | - |
| Calisiinae | *Aradacanthia heissi* | 135 | 2 | 3 | - | - |
| Aradinae | *Aradus compar* | 45 | 9 | 28 | 1342 | 56 |
| Carventinae | *Libiocoris heissi* | 52 | 2 | 88 | - | - |
| Aneurinae | *Aneurus sublobatus* | 48 | - | 62 | - | - |
|  | *Aneurus similis* | 45 | 2 | 20 | - | - |

-: not present.

**Supplementary Table S4.** Summary of Bayesian relaxed molecular clock analyses obtained under the autocorrelated Lognormal model and two data matrices for selected Pentatomomorpha clades with mean ages and 95% highest probability density intervals.

| **Taxa clade** | **PCGRNA** | **PCG12RNA** |
| --- | --- | --- |
| (Pentatomomorpha, Cimicomorpha) | 222 (210, 243) | 221 (211, 248) |
| Pentatomomorpha | 213 (207, 224) | 214 (207, 226) |
| (Pentatomoidea, Eutrichophora) | 181 (168, 194) | 188 (174, 201) |
| Aradoidea | 160 (143, 177) | 162 (140, 181) |
| Pentatomoidea | 128 (120, 131) | 128 (120, 131) |
| Eutrichophora | 147 (127, 163) | 148 (126, 166) |
| Coreoidea | 128 (108, 146) | 129 (108, 149) |
| (Pyrrhocoroidea, Lygaeoidea) | 135 (115, 153) | 138 (115, 158) |
| Lygaeoidea | 109 (89, 127) | 106 (85, 126) |
| Pyrrhocoroidea | 98 (78, 117) | 92 (70, 115) |
| (Aradinae, (Carventinae, (Mezirinae, Aneurinae))) | 125 (106, 144) | 134 (113, 155) |
| (Carventinae, (Mezirinae, Aneurinae)) | 104 (83, 125) | 112 (84, 125) |
| (Mezirinae, Aneurinae) | 98 (78, 118) | 105 (84, 125) |
| Mezirinae | 47 (33, 66) | 50 (34, 68) |
| Aneurinae | 64 (46, 83) | 67 (49, 85) |
|  |  |  |

**Supplementary Table S5.** Collection information of flat bugs newly sequenced in the present study.

| **Species** | **Locality** | **Time** | **Voucher specimen number** |
| --- | --- | --- | --- |
| *Aneurus similis* | Changjiang, Hainan (19°07' N, 109°06' E) | 16, October 2010 | VHem-501 |
| *Aneurus sublobatus* | Changjiang, Hainan (19°07' N, 109°06' E) | 16, October 2010 | VHem-502 |
| *Aradus compar* | Shennongjia, Hubei (31°42' N, 110°38' E) | 29, June 2009 | VHem-201 |
| *Libiocoris heissi* | Sanya, Hainan (18°44' N, 108°52' E) | 5, January 2010 | VHem-401 |

**Supplementary Table S6.** Taxa used in this study.

|  | **Suborder** | **Infraorder** | **Family** | **Species** | **GenBank number** |
| --- | --- | --- | --- | --- | --- |
| Outgroup |  |  |  |  |  |
| Hemiptera | Cimicomorpha | Reduvioidea | Reduviidae | *Agriosphodrus dohrni* | NC_015842 |
|  |  |  |  | *Peirates arcuatus* | NC_024264 |
|  |  | Miroidea | Miridae | *Apolygus lucorum* | NC_023083 |
|  |  |  |  | *Adelphocoris fasciaticollis* | NC_023796 |
|  |  | Naboidea | Nabidae | *Alloeorhynchus bakeri* | NC_016432 |
|  |  |  |  | *Gorpis humeralis* | NC_019593 |
| Ingroup |  |  |  |  |  |
| Hemiptera | Pentatomomorpha | Aradoidea | Aradidae | *Neuroctenus parus* | NC_012459 |
|  |  |  |  | *Aradacanthia heissi* | HQ441233 |
|  |  |  |  | *Brachyrhynchus hsiaoi* | NC_022670 |
|  |  |  |  | *Aradus compar* | present study |
|  |  |  |  | *Libiocoris heissi* | present study |
|  |  |  |  | *Aneurus sublobatus* | present study |
|  |  |  |  | *Aneurus similis* | present study |
|  |  | Pentatomoidea | Cydnidae | *Macroscytus subaeneus* | NC_012457 |
|  |  |  | Pentatomidae | *Nezara viridula* | NC_011755 |
|  |  |  |  | *Halyomorpha halys* | NC_013272 |
|  |  |  |  | *Dolycoris baccarum* | KM244699 |
|  |  |  | Plataspidae | *Coptosoma bifaria* | NC_012449 |
|  |  |  |  | *Megacopta cribraria* | NC_015342 |
|  |  |  | Tessaratomidae | *Eusthenes cupreus* | NC_022449 |
|  |  |  | Urostylidae | *Urochela quadrinotata* | NC_020144 |
|  |  |  | Dinidoridae | *Coridius chinensis* | JQ739179 |
|  |  | Lygaeoidea | Berytidae | *Yemmalysus parallelus* | NC_012464 |
|  |  |  | Colobathristidae | *Phaenacantha marcida* | NC_012460 |
|  |  |  | Malcidae | *Malcus inconspicuus* | NC_012458 |
|  |  |  | Geocoridae | *Geocoris pallidipennis* | NC_012424 |
|  |  |  | Lygaeidae | *Kleidocerys resedae resedae* | KJ584365 |
|  |  | Pyrrhocoroidea | Pyrrhocoridae | *Dysdercus cingulatus* | NC_012421 |
|  |  |  | Largidae | *Physopelta gutta* | NC_012432 |
|  |  | Coreoidea | Coreidae | *Hydaropsis longirostris* | NC_012456 |
|  |  |  | Alydidae | *Riptortus pedestris* | NC_012462 |
|  |  |  | Rhopalidae | *Stictopleurus subviridis* | NC_012888 |
|  |  |  |  | *Aeschyntelus notatus* | NC_012446 |

**Supplementary Table S7.** The best partitioning scheme selected by PartitionFinder for different data matrices.

| Data matrices | **Subset Partitions** | **Best Model** |
| --- | --- | --- |
| PCGR | P1: (*atp6*, *atp8*, *nad3*) | GTR+I+G |
| 10 partitions | P2: (*cox1*) | GTR+I+G |
|  | P3: (*cox2*, *cox3*, *cob*) | GTR+I+G |
|  | P4: (*nad1*) | GTR+I+G |
|  | P5: (*nad2*) | GTR+I+G |
|  | P6: (*nad4*) | GTR+I+G |
|  | P7: (*nad4L*, *nad5*) | GTR+I+G |
|  | P8: (*nad6*) | GTR+I+G |
|  | P9: (*srRNA*) | GTR+I+G |
|  | P10: (*lrRNA*) | GTR+I+G |
| PCG12R | P1: (*atp6*, *atp8*, *nad3*) | GTR+I+G |
| 9 partitions | P2: (*cox1*) | GTR+I+G |
|  | P3: (*cox2*, *cox3*, *cob*) | GTR+I+G |
|  | P4: (*nad1*, *nad4L*) | GTR+I+G |
|  | P5: (*nad2*) | GTR+I+G |
|  | P6: (*nad4*, *nad5*) | GTR+I+G |
|  | P7: (*nad6*) | GTR+I+G |
|  | P8: (*srRNA*) | GTR+I+G |
|  | P9: (*lrRNA*) | GTR+I+G |

**Supplementary Table S8.** Testing for best fit of various relaxed clock models in PhyloBayes.

| **Relaxed clock models** | **PCGRNA** | **PCG12RNA** |
| --- | --- | --- |
| UGAM vs Clock | 35.00 (31.28/35.76) | 26.25 (25.45/30.62) |
| Lognormal vs Clock | 50.46 (28.17/56.64) | 49.39 (10.22/55.23) |
| CIR vs Clock | 61.59 (12.00/67.98) | 56.93 (14.82/62.82) |
| Lognormal vs CIR | 0.63 (0.62 /0.76) | 1.092 (0.787/1.095) |

Values are the Bayes factors of variance-covariance matrix calculated for each of two datasets. The discrete error from which the Bayes factor has been derived is shown in brackets. Two autocorrelated relaxed clock models (CIR and Lognormal) have the similar Bayes factor values, better than the uncorrelated gamma model (UGAM). “-auto” analyses select the Lognormal as the best fitting model for both datasets.

**Supplementary Table S9.** Fossil calibrations with fossil taxonomic information, fossil age and references.

| **Fossils** | **Node assigned** | **Minimum**  **age (MYA)** | **Maximum**  **age (MYA)** | **References** |
| --- | --- | --- | --- | --- |
| *Paraknightia magnifica* | Pentatomomorpha+ Cimicomorpha | 208 | 251 | 1 |
| *Kerjiecoris oopsis* | Pentatomomorpha | 208 | 228 | 2 |
| *Cydnavites, Orienicydnus* | Pentatomoidea | 113 | 130 | 3 |
| *Asopus puncticollis* | Pentatomidae | 56 | 65 | 4 |

**Supplementary Figure S1**. Phylogenies of Pentatomomorpha inferred from two concatenated nucleotide datasets using maximum likelihood and Bayesian inference. Supports at nodes are bootstrap supports and Bayesian posterior probabilities.


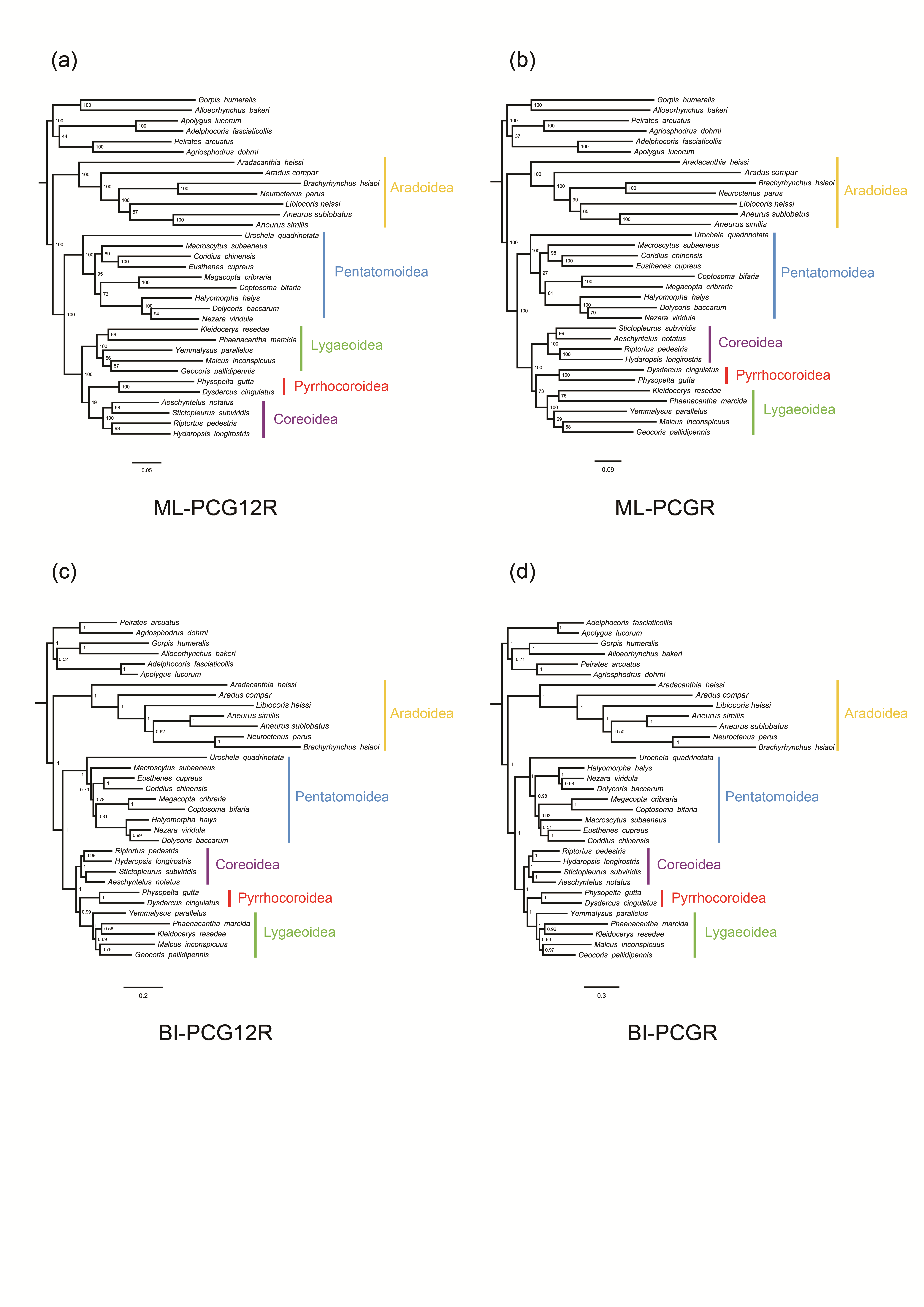


**Supplementary References**

1. Evans, J.W. Upper Permian Homoptera from New South Wales. *Rec. Aust. Mus.* **21,** 180-198 (1943).
2. Lin, Q.B. Late Triassic insect fauna from Toksun, Xinjiang. *Acta Pal. Sin.* **31,** 313-335 (1992).
3. Yao, Y.Z., Cai, W.Z. & Ren, D. The first fossil Cydnidae (Heteroptera: Pentatomoidea) from the Late Mesozoic of China. *Zootaxa* **1338,** 59–68 (2007).
4. Piton, E. Paléontologie du gisement Éocène de Menat (Puy-de-Dôme), flore et faune. *Mém Soc. His. Nat. Auvergne* **1,** 1–303 (1940).
